# Fosfomycin trometamol vs ciprofloxacin for antibiotic prophylaxis before transrectal ultrasonography-guided prostate biopsy: A meta-analysis of clinical studies

**DOI:** 10.1080/2090598X.2019.1592636

**Published:** 2019-04-11

**Authors:** Daniel Melecchi de Oliveira Freitas, Daniel M. Moreira

**Affiliations:** a Urologist at Hospital Moinhos de Vento and Nossa Senhora da Conceição Hospital, Porto Alegre, Brazil; b Department of Urology, University of Illinois at Chicago, Chicago, IL, USA

**Keywords:** Prostatic neoplasm, biopsy, fosfomycin, ciprofloxacin

## Abstract

**Objectives**: To perform a systematic review and meta-analysis of clinical studies to assess the comparative prophylactic effectiveness of fosfomycin trometamol (FMT) vs ciprofloxacin (CIP) in men who underwent transrectal ultrasonography-guided prostate needle biopsy (TRUS-PNB), as infectious complications are a major concern after TRUS-PNB and although fluoroquinolones are currently the first choice, an increase in resistance has raised the question about its recommendation and FMT is a broad-spectrum oral antibiotic with low bacterial resistance.

**Methods**: A systematic review was performed between January 1970 and June 2017 using the Web of Science, Scopus and PubMed databases to identify relevant studies. Preferred Reporting Items for Systematic Reviews and Meta-analysis criteria were used for article selection. Outcomes of interest were febrile and afebrile urinary tract infections (UTIs) and the presence of fluoroquinolone-resisitant (FQR)- or extended-spectrum β-lactamase (ESBL)-producing uropathogens in urinary cultures.

**Results**: Four studies including 2331 men were analysed; 1088 had FMT and 1243 CIP as antibiotic prophylaxis before TRUS-PNB. FMT prophylaxis resulted in significantly less afebrile (odds ratio [OR] 0.21, 95% confidence interval [CI] 0.12–0.38; *P* < 0.001) and febrile (OR 0.15, 95% CI 0.07–0.31; *P* < 0.001) UTIs than CIP. Amongst all urine cultures, patients in the FMT arm also had a significantly lower prevalence of FQR and ESBL (*E. coli* or *K. pneumoniae*) microorganisms when compared to the CIP group (OR 0.25, 95% CI 0.12–0.21, *P* = 0.001; and OR 0.24, 95% CI 0.10–0.58, *P* = 0.001, respectively).

**Conclusions**: Antibiotic prophylaxis with FMT before TRUS-PNB was associated with lower rates of infectious complications when compared to CIP.

**Abbreviations**: CIP: ciprofloxacin; ESBL: extended-spectrum β-lactamase; FMT: fosfomycin trometamol; FQR: fluoroquinolone-resisitant; OR: odds ratio; PRISMA: Preferred Reporting Items for Systematic Reviews and Meta-Analyses; TRUS-PNB: TRUS-guided prostate needle biopsy

## Introduction

UTI is one of the most common complications following TRUS-guided prostate needle biopsy (TRUS-PNB), with an incidence varying from 0.1% to 7% [1,2]. Recently, Nam et al. [3] reported that infectious complications were responsible for 70% of all hospitalisations in patients who underwent TRUS-PNB, leading to a considerable impact on health costs. Previous studies have found that the incidence of UTI after TRUS-PNB was associated with age, immunosuppression, chronic diseases, and previous use of antibiotics [4,5]. Moreover, the bacterial flora of the rectum and the type of antibiotic prophylaxis used has been correlated with infectious complications after TRUS-PNB [6]. Currently, fluoroquinolones are the most widely used antibiotic prophylaxis for TRUS-PNB [1]. However, several studies reported an increasing incidence of fluoroquinolone-resistant (FQR) uropathogens [6,7]. These findings urge the reconsideration of fluoroquinolones as the antibiotic of choice for TRUS-PNB prophylaxis.

Fosfomycin trometamol (FMT) is an antibiotic agent that acts by inhibiting the biosynthesis of peptidoglycans and has a wide spectrum against Gram-negative and Gram-positive microorganisms [8]. Its safety and efficacy have been confirmed in previous studies [9,10]. Furthermore, the bacterial resistance rate is extremely low (<3%) and cross-resistance with fluoroquinolones is rare [11]. Additionally, it has been shown to be effective against β-lactamase producing bacteria. For example, Pullukcu et al. [12], who studied the action of FMT in 54 patients with UTIs caused by extended-spectrum β-lactamase (ESBL)-producing *E. coli*, reported a treatment success rate of 94.3%. Multiple studies have evaluated the safety and efficacy of FMT as a prophylactic agent in patients undergoing TRUS-PNB with mixed results [13,14]. Thus, in the present study we sought to perform a systematic review and meta-analysis of the use of FMT in patients undergoing TRUS-PNB compared to ciprofloxacin (CIP) in preventing infectious complications after TRUS-PNB.

## Methods

### Evidence acquisition

**Figure 1.**
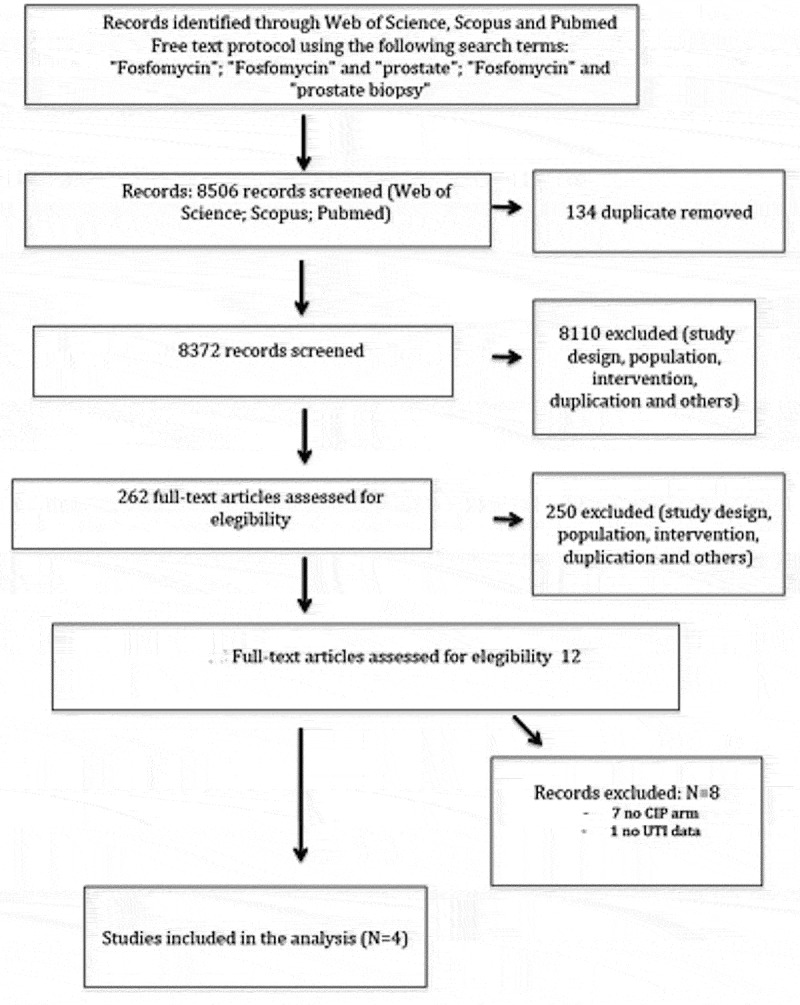
PRISMA flowchart.

After a systematic literature search including articles published between January 1970 and June 2017 using the Web of Science, Scopus and PubMed databases with the following relevant search terms: ‘fosfomycin’, ‘prostate’, ‘prostate biopsy’, we retrieved 8506 abstracts. After exclusion of 134 duplicates this resulted in 8372 abstracts. We retrieved a total of 262 abstracts selected for review based on the following criteria: study comparing FMT to other antibiotics for TRUS-PNB prophylaxis, English language, original research, and adult human subjects. Only published studies comparing FMT vs another antibiotic used as antibiotic prophylaxis before TRUS-PNB were included. Following the same criteria we carefully selected a total of 12 for full-text review. Of these, eight additional studies were excluded, seven due to absence of CIP in the control group and one due to absence of data about UTI, for a final study sample of four studies [15–18]. The final study sample was 1088 subjects in the FMT group and 1243 in the CIP group. Preferred Reporting Items for Systematic Reviews and Meta-Analyses (PRISMA) criteria (www.prismastatement.org) were used (Figure 1).

### Outcomes measured

Infectious complications were divided in two groups: afebrile and febrile UTIs. Afebrile UTI was defined as the presence of irritative urinary symptoms, e.g. dysuria, urgency or frequency, and pyuria (>10 leucocytes/high-power field; >5 leucocytes/high-power field in the Fahmy et al. [17] study) within 1 month after the biopsy. Febrile UTI was defined as the association amongst, irritative urinary symptoms, pyuria and fever >38°C within 1 month after the biopsy. Significant bacteriuria was defined as the presence of >10^5^ colony-forming units/mL of urine. Data were collected within 1 month of the biopsy.

### Types of intervention

Patients received FMT or CIP as preoperative antibiotic prophylaxis before TRUS-PNB.

### Statistical analysis

The main objective of the meta-analysis was to evaluate the infectious outcomes amongst patients who underwent TRUS-PNB comparing FMT to CIP as antibiotic prophylaxis. Outcomes considered included afebrile, febrile and FQR or ESBL UTIs. The presence of heterogeneity across studies was evaluated using the *I*^2^ statistic. Summary of effects for the outcomes evaluated were calculated as odds ratios (ORs) and 95% CIs comparing FMT to CIP using a random effects model given the methodological variability across studies, e.g. different antibiotic dose and schedule. Risk of bias was assessed using the Cochrane Risk of Bias Tool for clinical trials and the Newcastle-Ottawa Scale for observational studies [19]. All statistical analyses were performed using Review Manager Software (RevMan, version 5.1, Cochrane Collaboration, Oxford, UK). A *P* < 0.05 was considered to indicate statistical significance.

## Results

In all, 2331 men were included in the final analysis. Amongst these, 1088 had FMT and 1243 CIP as antibiotic prophylaxis. Of the four studies included in the meta-analysis, Cai et al. [18] and Ongün et al. [15] were retrospective; and Sen et al. [16] and Fahmy et al. [17] were prospective randomised studies. All studies included men who underwent TRUS-PNB due to elevated PSA levels and/or had abnormal DREs. The Sen et al. [16] and Ongün et al. [15] studies reported PSA threshold levels of >2.5 ng/dL as an indication for biopsy, whilst Cai et al. [18] and Fahmy et al. [17] did not report PSA threshold levels data. TRUS was performed in lithotomy or lateral decubitus under local anaesthesia. Prostate specimens (10–14 cores) were taken using an automated biopsy gun with a 16- or 18-G needle. Men with positive urinary cultures and using indwelling urethral catheters were excluded. Ongün et al. [15] also excluded men with a previous history of urinary tract surgery in the last month or who had undergone saturation biopsy (24 cores). Fahmy et al. [17] and Sen et al. [16] excluded men with a previous history of UTI, whilst Cai et al. [18] excluded only those with previous CIP or FMT-resistant UTIs. Moreover, in the Cai et al. [18] study men diagnosed with urinary tract structural abnormalities and with a Charlson Comorbidity Index >3 were also excluded. Men with previous use of any kind of antibiotics 1 month before TRUS-PNB were also excluded in the Sen et al. [16] study. All four studies reported UTI complications as described previously except for Fahmy et al. [17] where the diagnosis of UTI was based on a positive urine culture. All studies used the same 3 g oral FMT dosage before the biopsy [16,17]. However, Cai et al. [18] used another FMT dose 24 h after the biopsy. The CIP dose and schedule varied across studies. In the Sen et al. [16] study, patients received a single of 500 mg oral CIP 60 min before the biopsy; whilst in the Fahmy et al. [17] study, 500 mg oral metronidazole and 500 mg CIP were given 1 h prior to TRUS-PNB followed by CIP and metronidazole twice a day for 3 days. The other two studies reported the use of 500 mg oral CIP twice daily for 5 days starting 1 day before the biopsy.

Baseline patient characteristics are presented in Table 1 [15–18]. There was no difference in age between the FMT and CIP groups, at a mean (SD) of 65.7 (7.67) vs 64.7 (7.69) years, respectively. Similarly, prostate volume (mL) and PSA levels (ng/mL) were comparable between the treatment groups in the three studies that reported these variables. Men in the FMT groups were more likely to have had a prior biopsy but this did not reach statistical significance.

**Table 1.** FMT vs CIP: summary data of the four clinical studies.

Reference	Number of cases	Study period	Study design	Intervention	Setting
Ongün et al. [15]	104 FMT 406 CIP	2010–2011	Retrospective cohort	CIP 500 mg orally twice/day for 5 days vs FMT 3000 mg oral single dose	Turkey
Cai et al. [18]	632 FMT 477 CIP	2015	Retrospective cohort	CIP 500 mg oral twice/day for 5 days vs FMT 3000 mg orally with another dose 24 h after	Italy, Germany and Norway
Fahmy et al. [17]	202 FMT 210 CIP	2012–2015	RCT	CIP 500 mg + MTZ 500 mg orally twice/day for 3 days vs FMT 3000 mg oral single dose	Egypt
Sen et al. [16]	150 FMT 150 CIP	2014–2015	RCT	CIP 500 mg orally vs FMT 3000 mg orally Both single dose	Turkey

MTZ, metronidazole; RCT, randomised controlled trial.

**Figure 2.**
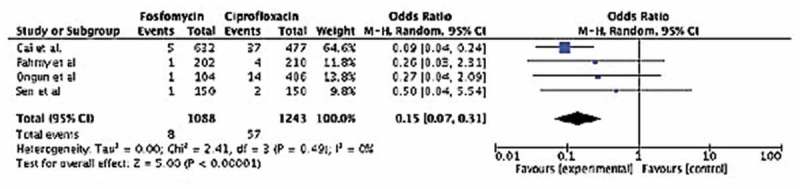
Febrile UTI.

Amongst all men in the four studies, afebrile UTI was diagnosed in 103 (4.4%). There was no significant heterogeneity across the studies (*I*^2^ = 0%, *P* = 0.61). The presence of afebrile UTI was significantly lower in the FMT group compared to the CIP group (OR 0.21, 95% CI 0.12–0.38; *P* < 0.001; Figure 2).

**Figure 3.**
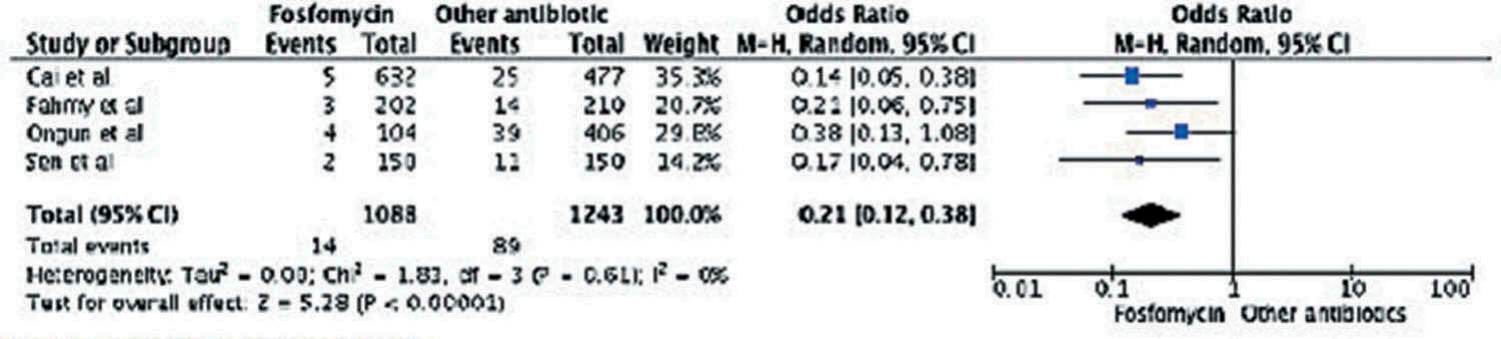
Afebrile UTI.

A total of 65 men (2.8%) were diagnosed with febrile UTIs, eight (12.3%) in the FMT group and 57 (87.7%) in the CIP group (Figure 3). This represents 0.7% of all FMT and 4.6% of all CIP subjects. There was no significant heterogeneity across studies (*I*^2^ = 0%, *P* = 0.46). The odds of febrile UTI was significantly less in men who were in the FMT group (OR 0.15, 95% CI 0.07–0.31; *P* < 0.001). Amongst all urine cultures obtained, FQR and ESBL (*E. coli* or *K. pneumoniae*) microorganisms were found more frequently in the CIP group (OR 0.25, 95% CI 0.12–0.21, *P* = 0.001; and OR 0.24, 95% CI 0.10–0.58, *P* = 0.001, respectively).

## Discussion

Currently, fluoroquinolones are the first choice of antibiotic prophylaxis for TRUS-PNB, as their safety and efficacy have been tested in several clinical trials [20,21]. However, the increasing prevalence of CIP-resistant bacteria in several countries is a matter of concern [22]. Recent studies have shown that the incidence of bacteria resistant to CIP is, on average, 25%, but in some cases, it can be as high as 70% [23,24]. This led to research investigating alternative antibiotics for TRUS-PNB prophylaxis including FMT, an oral broad-spectrum antibiotic with a very low microbial resistance and cross-resistance to CIP [8,25]. Unfortunately, previous studies evaluating the role of FMT in TRUS-PNB prophylaxis were underpowered and showed mixed results. Therefore, we performed a systematic review and meta-analysis comparing FMT to CIP in men undergoing TRUS-PNB. We found that FMT was associated with a lower incidence of febrile and afebrile UTIs in these men.

Previously, two randomised clinical trials have compared FMT vs CIP [16,17]. Sen et al. [16] studied 300 men allocated to receive either 3 g oral FMT the night before the biopsy or 500 mg CIP 60 min before the TRUS-PNB. Infectious complications were more common in the CIP group when compared to the FMT group (*P* = 0.03). However, whilst afebrile UTI was significantly more frequent in the CIP arm (1.3% vs 6.0%), the incidence of febrile UTI was not different between the groups. In that study, 45.5% of men with UTIs who received CIP had *E. coli* or *K. pneumoniae* resistant to fluoroquinolones, whilst in the FMT group the incidence of CIP-resistant infection was much lower [16]. More recently, Fahmy et al. [17] randomised 412 men undergoing TRUS-PNB to receive either 3 g oral FMT or 500 mg oral CIP with metronidazole 500 mg 1 h before the intervention and then twice daily for 3 days. In that study, the incidence of febrile and afebrile UTIs was higher in the CIP group (1.98%) than in the FMT group (8.57%, *P* = 0.001). Interestingly, the rate of FQR infection was four-times more frequent in the CIP group compared to the FMT group (1.48% vs 6.19%). Moreover, all strains that were resistance to CIP were also EBSL-producing *E. coli* and *K. pneumoniae* [17].

Cai et al. [18], in an observational cohort study, included data from 1109 men who had received 3 g oral FMT before and 3 g 24 h after the biopsy vs 500 mg CIP starting 1 day before the biopsy and continued twice a day for 5 days. The rate of UTI was significantly higher in the CIP group than in the FMT group (*P* < 0.001). In that study, the presence of FQR uropathogens was not different between groups. Ongün et al. [15] analysed 640 men who had undergone TRUS-PNB and received 3 g oral FMT the night before the biopsy or 500 mg CIP twice daily for 5 days. The incidence of UTI was not different between the groups. However, in that article the prevalence of FQR bacteria in febrile UTI cases was >60% and was present only in the CIP group [15].

Our present results show that FMT was associated with a lower incidence of infectious complications (both afebrile and febrile UTI). The most plausible biological explanation for such a finding is the lower bacterial resistance associated with FMT. This is corroborated by studies showing that bacteria in rectal flora are typically sensitive to FMT and the worldwide increase in bacterial resistance to fluoroquinolones [23–25]. Moreover, Liss et al. [26] reported that the main cause of UTI following transrectal procedures is the presence of FQR bacteria, usually *E. coli*. Thus, given the high prevalence of CIP resistance and the lower efficacy of CIP in preventing TRUS-PNB-related UTIs, a safer alternative to CIP for TRUS-PNB prophylaxis should be strongly considered. Given some studies have shown that infectious complications are the most common cause of hospitalisation after TRUS-PNB, any efforts to reduce UTI in this setting can lead to a substantial reduction in morbidity and suffering. As such, FMT seems to be a safe and efficacious antibiotic prophylaxis alternative to CIP with excellent tolerability, which can minimise complications and costs associated with TRUS-PNB.

Our present meta-analysis has several limitations. First, the dosage of antibiotics amongst the studies was heterogeneous. Moreover, Sen et al. [16] and Fahmy et al. [17] excluded men who received antibiotics within a month prior to the biopsy. Second, the studies included in the meta-analysis were performed in multiple countries with likely diverse microbiological colonic flora. Although this increases the heterogeneity of our present sample, it increases the external validity and applicability of the four studies results. Third, men with certain comorbidities, e.g. diabetes, were excluded in some studies, which could lead to a potential selection bias. Fourth, the number of biopsy cores, bowel preparation, cleansing enema, and needle disinfection were not controlled or standardised in the studies.

In conclusion, in a meta-analysis of four studies evaluating FMT vs CIP in preventing infectious complications amongst men undergoing TRUS-PNB, FMT was associated with lower febrile and afebrile UTI rates. The increased incidence of FQR bacteria in urinary cultures strongly suggests that alternatives to CIP should be studied to mitigate infectious complications. FMT seems a good option for TRUS-PNB prophylaxis, potentially reducing the incidence of infectious complications.
